# Model for Aqueous Polymer Solutions with Damping Term: Solvability and Vanishing Relaxation Limit

**DOI:** 10.3390/polym14183789

**Published:** 2022-09-10

**Authors:** Evgenii S. Baranovskii, Mikhail A. Artemov

**Affiliations:** Department of Applied Mathematics, Informatics and Mechanics, Voronezh State University, 394018 Voronezh, Russia

**Keywords:** non-Newtonian fluid, aqueous polymer solutions, relaxation viscosity, damping, nonlinear partial differential equations, full weak solution, existence theorem, monotonicity, compactness, acute angle theorem

## Abstract

The main aim of this paper is to investigate the solvability of the steady-state flow model for low-concentrated aqueous polymer solutions with a damping term in a bounded domain under the no-slip boundary condition. Mathematically, the model under consideration is a boundary value problem for the system of strongly nonlinear partial differential equations of third order with the zero Dirichlet boundary condition. We propose the concept of a full weak solution (velocity–pressure pair) in the distributions sense. Using the method of introduction of auxiliary viscosity, the acute angle theorem for generalized monotone nonlinear operators, and the Krasnoselskii theorem on the continuity of the superposition operator in Lebesgue spaces, we obtain sufficient conditions for the existence of a full weak solution satisfying some energy inequality. Moreover, it is shown that the obtained solutions of the original problem converge to a solution of the steady-state damped Navier–Stokes system as the relaxation viscosity tends to zero.

## 1. Introduction

Many real fluids and fluid-like materials cannot be adequately modeled by the classical Navier–Stokes equations [1,2,3,4]. Such fluids are said to be *non-Newtonian*. Examples are polymeric liquids, oil, blood, cements, bitumen, concrete, and suspension of river sand, as well as some liquids arising in food processing. The mathematical study of flow models for non-Newtonian fluids is very important in various scientific and technological applications. It is well known that most non-Newtonian materials have a complex (sometimes unpredictable) nature of behavior, and their models often produce significant difficulties in mathematical handling. One such model is the model for the motion of low-concentrated aqueous polymer solutions [5,6,7,8]:

(1) $$\left\{\begin{array}{c} \rho \left(\partial_{t}\vec{v}+\left(\vec{v}\cdot \nabla \right)\vec{v}\right)-\mathrm{div}\left[\mu D\vec{v}+\alpha \partial_{t}D\vec{v}+\alpha \left(\vec{v}\cdot \nabla \right)D\vec{v}\right]+\nabla p=\rho \vec{F}+\gamma {\left|\vec{v}\right|}^{\beta -2}\vec{v},\\ \mathrm{div}\;\vec{v}=0, \end{array}\right.$$

where, to fix the notation,

- *t* denotes time;
- ρ is the fluid density, ρ>0;
- $\vec{v}$ is the flow velocity;
- *p* is the pressure;
- the symbol ∇ denotes the gradient with respect to the space variables $x_{1},\cdots ,x_{d}$, that is, $\nabla \overset{\mathrm{def}}{=}\left(\frac{\partial }{\partial x_{1}},\cdots ,\frac{\partial }{\partial x_{d}}\right)$, where d=2 or 3;
- $D\vec{v}=\left(D_{ij}\vec{v}\right)$ is the strain rate tensor corresponding to the vector field $\vec{v}$,
  $$D_{ij}\vec{v}\overset{\mathrm{def}}{=}\frac{1}{2}\left(\frac{\partial v_{i}}{\partial x_{j}}+\frac{\partial v_{j}}{\partial x_{i}}\right),\;i,j=1,\cdots ,d;$$
- the operator div is defined as follows:
  $$\mathrm{div}\;\vec{v}\overset{\mathrm{def}}{=}\sum_{i=1}^{d}\frac{\partial v_{i}}{\partial x_{i}},\;\mathrm{div}\;M\overset{\mathrm{def}}{=}\left(\sum_{i=1}^{d}\frac{\partial M_{i1}}{\partial x_{i}},\cdots ,\sum_{i=1}^{d}\frac{\partial M_{id}}{\partial x_{i}}\right),$$
  for a vector function $\vec{v}=\left(v_{1},\cdots ,v_{d}\right)$ and a matrix-valued function $M=(M_{ij})$;
- μ and α denote the dynamic viscosity and the relaxation viscosity, respectively (μ>0 and α≥0);
- $\vec{F}$ is the external forces field;
- the nonlinear damping term $\gamma |\vec{v}{|}^{\beta -2}\vec{v}$ in the balance of linear momentum realizes an absorption if γ<0, and a nonlinear source if γ>0 (see [9,10]);
- β is the damping exponent, β>1.

The mathematical model (1) has been confirmed by experimental studies; in particular, it is considered as an appropriate model for the motion of aqueous solutions of polyethylenoxide, polyacrylamide, and guar gum [11,12]. Note that the limit case α=0 corresponds to the incompressible Navier–Stokes equations with damping (in the literature, these equations are often called the convective Brinkman–Forchheimer equations), which describe a Newtonian fluid flow through a porous medium [13,14].

There is extensive literature devoted to the analysis of the well-posedness of the aqueous polymer solutions model and its modifications (see, e.g., [6,15,16,17,18,19,20] and the references therein). However, all results are obtained for the case γ=0, and the main questions concerning the solvability and properties of solutions to system (1) are still open.

Some researchers have considered a simplified version of model (1), assuming that

(2) $$\mathrm{div}\left[\left(\vec{v}\cdot \nabla \right)D\vec{v}\right]\equiv \vec{0}.$$

In this case, system (1) reduces to the so-called damped Kelvin–Voigt equations

(3) $$\left\{\begin{array}{c} \rho \left(\partial_{t}\vec{v}+\left(\vec{v}\cdot \nabla \right)\vec{v}\right)-\mathrm{div}\left[\mu D\vec{v}+\alpha \partial_{t}D\vec{v}\right]+\nabla p=\rho \vec{F}+\gamma {\left|\vec{v}\right|}^{\beta -2}\vec{v},\\ \mathrm{div}\;\vec{v}=0, \end{array}\right.$$

which have more “good” mathematical properties in comparison with (1). Korpusov and Sveshnikov [21] proved the local-in-time unique solvability of an initial boundary value problem for system (3) with γ=−1 and β=4 (a cubic source) in the weak formulation. They also obtained some conditions on the initial velocity field, which ensure that the solution blows up in finite time. These results were extended and improved by Antontsev and Khompysh in [9]. The next investigation of this model was connected with the consideration of the situation when both the viscous and relaxation parts of the stress tensor are given by distinct power-laws, and the momentum equation is perturbed by an anisotropic damping term $\sum_{i=1}^{d}\gamma_{i}{\left|\vec{v}\right|}^{\beta_{i}-2}\vec{v}$ (see [22]). Let us mention also the paper [23], in which, by using the m-accretive quantization of linear and nonlinear operators, the existence and uniqueness of a strong solution to (3) was established under the condition that γ<0 and β≥2. Moreover, in this work, global and exponential attractors were studied for the corresponding dynamical system.

The passing from the original system (1) to (3) solves the main mathematical difficulties in studying the damped model for aqueous polymer solutions, but, from the physical point of view, it would be more interesting not to use the simplifying assumption (2) and keep all nonlinear terms in the motion equations. Motivated by this, in the present paper, we investigate the steady-state version (that is, the flow velocity $\vec{v}$, the pressure *p*, and the external force $\vec{F}$ are assumed to be time-independent) of model (1) in a bounded domain $\Omega \subset R^{d}$, d=2,3, under the no-slip condition on the boundary ∂Ω:

(4) $$\left\{\begin{array}{cc} \rho \left(\vec{v}\cdot \nabla \right)\vec{v}-\mathrm{div}\left[\mu D\vec{v}+\alpha \left(\vec{v}\cdot \nabla \right)D\vec{v}\right]+\nabla p=\rho \vec{F}+\gamma {\left|\vec{v}\right|}^{\beta -2}\vec{v}\; & \mathrm{in}\;\Omega ,\\ \mathrm{div}\;\vec{v}=0\; & \mathrm{in}\;\Omega ,\\ \vec{v}=\vec{0}\; & \mathrm{on}\;\partial \Omega . \end{array}\right.$$

The main aim of our work is to prove the solvability of problem (4) in the weak formulation. We introduce the concept of a full weak solution, which is defined as a pair $\left(\vec{v},p\right)$ satisfying the governing equations in the distributions sense. Moreover, we derive energy estimates for solutions and study the behavior of the flow velocity as α→0. Namely, using an energy inequality independent of α, we show the convergence of the weak solutions of (4) to a weak solution of the damped Navier–Stokes system

(5) $$\left\{\begin{array}{cc} \rho \left(\vec{v}\cdot \nabla \right)\vec{v}-\frac{\mu }{2}\nabla^{2}\vec{v}+\nabla p=\rho \vec{F}+\gamma {\left|\vec{v}\right|}^{\beta -2}\vec{v}\; & in\Omega ,\\ \mathrm{div}\;\vec{v}=0\; & in\Omega ,\\ \vec{v}=\vec{0}\; & on\partial \Omega , \end{array}\right.$$

when the relaxation viscosity α tends to zero.

To overcome the difficulties in the mathematical handling of the boundary value problems for the aqueous polymer solutions model, one can use the following two approaches:

(i) The method of introduction of auxiliary viscosity [6] (see also [24,25]), which involves a regularization of the original equations by extra terms with a small parameter ε and solving the regularized problem with the consequent passing to the limit as ε→0.
(ii) The modified Faedo–Galerkin scheme with a special basis [19,26].

The first approach is suitable only for solving problems with the zero Dirichlet boundary condition, while the second one is more universal and can be applied to slip problems too. Since in this paper we deal with the no-slip condition on solid walls of the flow domain, the method of introduction of auxiliary viscosity will be used to construct a solution of system (4). In order to establish the solvability of the corresponding regularized problem, we interpret it as an operator equation with a so-called ${\left(S\right)}_{+}$-operator [27,28] and apply the acute angle theorem (Proposition 3).

The outline of the paper is as follows. In the next section, we introduce the notation and function spaces. In Section 3, the concept of full weak solutions of problem (4) is given (Definition 1). Here, we also formulate our main results (Theorem 1). Section 4 is devoted to obtaining auxiliary results, which are needed for proving Theorem 1. Finally, in Section 5, we prove the main results of this work.

## 2. Preliminaries: Notation and Function Spaces

For vectors $\vec{a},\vec{b}\in R^{d}$ and matrices $A,B\in R^{d\times d}$, by $\vec{a}\cdot \vec{b}$ and A:B we denote the scalar products, respectively:

$$\vec{a}\cdot \vec{b}\overset{\mathrm{def}}{=}\sum_{i=1}^{d}a_{i}b_{i},\;A:B\overset{\mathrm{def}}{=}\sum_{i,j=1}^{d}A_{ij}B_{ij}.$$

The Euclidean norm |·| is defined as follows:

$$|\vec{a}|\overset{\mathrm{def}}{=}{\left(\vec{a}\cdot \vec{a}\right)}^{1/2},\;\left|A\right|\overset{\mathrm{def}}{=}{\left(A:A\right)}^{1/2}.$$

Let $E_{1}$ and $E_{2}$ be Banach spaces. By $L(E_{1},E_{2})$ we denote the space of all bounded linear mappings from $E_{1}$ into $E_{2}$.

As usual, the strong (weak) convergence in a Banach space is denoted by → (⇀).

We use the standard notation for the Lebesgue spaces $L^{s}\left(\Omega \right)$, s∈[1,∞) and the Sobolev spaces $H^{k}\left(\Omega \right)\overset{\mathrm{def}}{=}W^{k,2}\left(\Omega \right)$, k∈N (see [29,30] for details).

Let

$$\begin{array}{cc} D(\Omega ) & \overset{\mathrm{def}}{=}\left\{\vec{\phi }:\Omega \to R:\;\vec{\phi }\in C^{\infty }\left(\Omega \right),\;\mathrm{supp}\;\vec{\phi }\subset \Omega \right\},\\ D^{\prime }\left(\Omega \right) & \overset{\mathrm{def}}{=}\;\mathrm{the}\;\mathrm{dual}\;\mathrm{space}\;\mathrm{of}\;D\left(\Omega \right),\;\text{i.e.,}\;\mathrm{the}\;\mathrm{space}\;\mathrm{of}\;\mathrm{distributions}\;\mathrm{in}\;\Omega ,\\ H_{0}^{1}\left(\Omega \right) & \overset{\mathrm{def}}{=}\;\mathrm{the}\;\mathrm{closure}\;\mathrm{of}\;D\left(\Omega \right)\;\mathrm{in}\;\mathrm{the}\;\mathrm{Sobolev}\;\mathrm{space}\;H^{1}\left(\Omega \right),\\ H^{-1}\left(\Omega \right) & \overset{\mathrm{def}}{=}\;\mathrm{the}\;\mathrm{dual}\;\mathrm{space}\;\mathrm{of}\;H_{0}^{1}\left(\Omega \right). \end{array}$$

Let X(Ω) denote any of the classes introduced above (D(Ω), $L^{s}\left(\Omega \right)$, $H^{k}\left(\Omega \right)$, etc.). We shall use the notation $X{\left(\Omega \right)}^{n}$ for the Cartesian product of *n* spaces X(Ω).

The symbol ↪ denotes a continuous imbedding, while ↪↪ denotes a compact imbedding.

Note that the following imbeddings hold (see, e.g., [29], Chapter VI):

(6) $$\begin{array}{cc} \; & H^{1}\left(\Omega \right)↪↪L^{s}\left(\Omega \right),\;\;\forall \;s\in S\left(d\right), \end{array}$$

(7) $$\begin{array}{cc} \; & H^{2}\left(\Omega \right)↪↪C\left(\bar{\Omega }\right), \end{array}$$

(8) $$\begin{array}{cc} \; & H^{3}\left(\Omega \right)↪↪H^{2}\left(\Omega \right), \end{array}$$

where the set-valued map S:{2,3}⊸R is defined as follows:

$$\begin{array}{c} S\left(d\right)\overset{\mathrm{def}}{=}\left\{\begin{array}{cc} \left[1,+\infty \right) & \mathrm{if}d=2,\\ \left[1,6\right) & \mathrm{if}d=3. \end{array}\right. \end{array}$$

Assuming that

$$D_{\mathrm{sol}}{\left(\Omega \right)}^{d}\overset{\mathrm{def}}{=}\left\{\vec{\phi }\in D{\left(\Omega \right)}^{d}:\;\mathrm{div}\;\vec{\phi }=0\right\},$$

we define spaces of solenoidal vector functions:

$$\begin{array}{cc} V^{0}\left(\Omega \right) & \overset{\mathrm{def}}{=}\;\mathrm{the}\;\mathrm{closure}\;\mathrm{of}\;\mathrm{the}\;\mathrm{set}\;D_{\mathrm{sol}}{\left(\Omega \right)}^{d}\;\mathrm{in}\;\mathrm{the}\;\mathrm{Lebesgue}\;\mathrm{space}\;L^{2}{\left(\Omega \right)}^{d},\\ V^{k}\left(\Omega \right) & \overset{\mathrm{def}}{=}\;\mathrm{the}\;\mathrm{closure}\;\mathrm{of}\;\mathrm{the}\;\mathrm{set}\;D_{\mathrm{sol}}{\left(\Omega \right)}^{d}\;\mathrm{in}\;\mathrm{the}\;\mathrm{Sobolev}\;\mathrm{space}\;H^{k}{\left(\Omega \right)}^{d},\;k\in N. \end{array}$$

Sometimes it will be helpful to use the Helmholtz–Weyl decomposition for vector functions from $L^{2}{\left(\Omega \right)}^{d}$ into a divergence-free part and a gradient part (see [30], Chapter IV):

$$L^{2}{\left(\Omega \right)}^{d}=V^{0}\left(\Omega \right)⊕\nabla H^{1}\left(\Omega \right),$$

where the symbol ⊕ denotes the orthogonal sum and

$$\nabla H^{1}\left(\Omega \right)\overset{\mathrm{def}}{=}\left\{\nabla \xi :\;\xi \in H^{1}\left(\Omega \right)\right\}.$$

The orthogonal projection $P_{V^{0}\left(\Omega \right)}$ from the space $L^{2}{\left(\Omega \right)}^{d}$ into the subspace $V^{0}\left(\Omega \right)$ is known as the Leray projection.

We introduce the scalar product on the space $V^{1}\left(\Omega \right)$ and the associated norm as follows:

$${\left(\vec{v},\vec{w}\right)}_{V^{1}\left(\Omega \right)}\overset{\mathrm{def}}{=}\int_{\Omega }D\vec{v}:D\vec{w}\;dx,\;{∥\vec{v}∥}_{V^{1}\left(\Omega \right)}\overset{\mathrm{def}}{=}{\left(\vec{v},\vec{v}\right)}_{V^{1}\left(\Omega \right)}^{1/2}.$$

By using Korn’s inequality

$$∥\vec{v}{∥}_{H^{1}{\left(\Omega \right)}^{d}}\leq C\left(\Omega \right){∥D\vec{v}∥}_{L^{2}{\left(\Omega \right)}^{d\times d}},\;\forall \;\vec{v}\in H_{0}^{1}{\left(\Omega \right)}^{d},$$

where C(Ω) is a positive constant (see, e.g., [31], Chapter I, Section 2.2), it is easily shown that the norm ${∥\cdot ∥}_{V^{1}\left(\Omega \right)}$ is equivalent to the standard $H^{1}{\left(\Omega \right)}^{d}$-norm.

For any integer m≥1, one can define the operator ∇ to the power of 2m and 2m+1 as follows:

$$\nabla^{2m}\overset{\mathrm{def}}{=}\nabla^{2m-1}\cdot \nabla ,\;\nabla^{2m+1}\overset{\mathrm{def}}{=}\nabla \left(\nabla^{2m}\right).$$

We introduce the scalar product on the space $V^{3}\left(\Omega \right)$ and the associated norm by the formulas:

$${\left(\vec{v},\vec{u}\right)}_{V^{3}\left(\Omega \right)}\overset{\mathrm{def}}{=}\int_{\Omega }\nabla^{3}\vec{v}:\nabla^{3}\vec{u}\;dx+\int_{\Omega }\nabla^{2}\vec{v}\cdot \nabla^{2}\vec{u}\;dx,\;{∥\vec{v}∥}_{V^{3}\left(\Omega \right)}\overset{\mathrm{def}}{=}{\left(\vec{v},\vec{v}\right)}_{V^{3}\left(\Omega \right)}^{1/2}.$$

**Lemma** **1.** *Let $\partial \Omega \in C^{3}$. Then the norms ${∥\cdot ∥}_{V^{3}\left(\Omega \right)}$ and ${∥\cdot ∥}_{H^{3}{\left(\Omega \right)}^{d}}$ are equivalent; that is, there exist positive constants $C_{1}\left(\Omega \right)$ and $C_{2}\left(\Omega \right)$ such that*

(9) $$C_{1}\left(\Omega \right)∥\vec{v}{∥}_{H^{3}{\left(\Omega \right)}^{d}}\leq ∥\vec{v}{∥}_{V^{3}\left(\Omega \right)}\leq C_{2}\left(\Omega \right){∥\vec{v}∥}_{H^{3}{\left(\Omega \right)}^{d}},\;\forall \vec{v}\in V^{3}\left(\Omega \right).$$

**Proof.**  The right inequality in the chain (9) immediately follows from the definition of the $H^{3}{\left(\Omega \right)}^{d}$-norm.

Using the well-known results concerning the properties of solutions to the Stokes equations with the zero Dirichlet boundary condition (see, e.g., [32], Chapter I, Section 2), it is easily shown that

(10) $$∥\vec{v}{∥}_{H^{3}{\left(\Omega \right)}^{d}}\leq {\tilde{C}}_{1}\left(\Omega \right){∥P_{V^{0}\left(\Omega \right)}\left(\nabla^{2}\vec{v}\right)∥}_{H^{1}{\left(\Omega \right)}^{d}},\;\forall \vec{v}\in V^{3}\left(\Omega \right),$$

with a positive constant ${\tilde{C}}_{1}\left(\Omega \right)$.

Taking into account (10) and the following equality

$$P_{V^{0}\left(\Omega \right)}\left(\nabla^{2}\vec{\phi }\right)=\nabla^{2}\vec{\phi },\;\forall \vec{\phi }\in D_{\mathrm{sol}}{\left(\Omega \right)}^{d},$$

we obtain

$$∥\vec{\phi }{∥}_{H^{3}{\left(\Omega \right)}^{d}}\leq {\tilde{C}}_{1}\left(\Omega \right){∥\nabla^{2}\vec{\phi }∥}_{H^{1}{\left(\Omega \right)}^{d}},\;\forall \vec{\phi }\in D_{\mathrm{sol}}{\left(\Omega \right)}^{d}.$$

Moreover, since the set $D_{\mathrm{sol}}{\left(\Omega \right)}^{d}$ is dense in the space $V^{3}\left(\Omega \right)$, the last equality remains valid if we replace $\vec{\phi }$ by an arbitrary vector function $\vec{v}$ belonging to $V^{3}\left(\Omega \right)$:

$$∥\vec{v}{∥}_{H^{3}{\left(\Omega \right)}^{d}}\leq {\tilde{C}}_{1}\left(\Omega \right){∥\nabla^{2}\vec{v}∥}_{H^{1}{\left(\Omega \right)}^{d}},\;\forall \vec{v}\in V^{3}\left(\Omega \right).$$

This implies the left inequality in (9) with $C_{1}\left(\Omega \right)=1/{\tilde{C}}_{1}\left(\Omega \right)$. Thus, Lemma 1 is proved. □

**Remark** **1.** *In view of* (8)*, we have $V^{3}\left(\Omega \right)↪↪V^{2}\left(\Omega \right)$.*

## 3. Definition of Full Weak Solutions and Main Results

Using arguments similar to the ones in the proof of Lemma 2 from [33], it can easily be checked that the following statement is true.

**Proposition** **1** (Energy equality)**.** *If a pair $\left(\vec{v},p\right)$ is a classical solution of problem* (4)*, then*

$$\mu \int_{\Omega }|D\vec{v}{|}^{2}\;dx=\rho \int_{\Omega }\vec{F}\cdot \vec{v}\;dx+\gamma \int_{\Omega }{\left|\vec{v}\right|}^{\beta }\;dx.$$

The question of the existence of classical solutions to problem (4) is delicate, especially in the case when the forcing term $\vec{F}$ is non-smooth and/or has a large norm. Therefore, it is reasonable to move from classical solutions to weak solutions, which can be constructed for model data from a wide class. Proposition 1 prompts how to define a weak solution without losing the energy estimate.

Let us introduce the set-valued map B:{2,3}×R⊸R by the formula

$$\begin{array}{c} B\left(d,\gamma \right)\overset{\mathrm{def}}{=}\left\{\begin{array}{cc} \left(1,+\infty \right) & \mathrm{if}\;d=2\;\mathrm{and}\;\gamma \leq 0,\\ \left(1,2\right) & \mathrm{if}\;d=2\;\mathrm{and}\;\gamma >0,\\ \left(1,7/2\right) & \mathrm{if}\;d=3\;\mathrm{and}\;\gamma \leq 0,\\ \left(1,2\right) & \mathrm{if}\;d=3\;\mathrm{and}\;\gamma >0 \end{array}\right. \end{array}$$

and assume that

(11) $$\begin{array}{c} \left\{\begin{array}{c} \partial \Omega \in C^{3},\\ \vec{F}\in H^{-1}{\left(\Omega \right)}^{d},\\ \beta \in B(d,\gamma ). \end{array}\right. \end{array}$$

**Definition** **1** (Full weak solution)**.** *We say that a pair $\left(\vec{v},p\right)\in V^{1}\left(\Omega \right)\times D^{\prime }\left(\Omega \right)$ is a full weak solution of problem* (4) *if*

(12) $$\begin{array}{cc} \; & -\rho \sum_{i=1}^{d}\int_{\Omega }v_{i}\vec{v}\cdot \frac{\partial \vec{\varphi }}{\partial x_{i}}\;dx+\mu \int_{\Omega }D\vec{v}:D\vec{\varphi }\;dx-\alpha \sum_{i=1}^{d}\int_{\Omega }v_{i}D\vec{v}:\frac{\partial D\vec{\varphi }}{\partial x_{i}}\;dx\\ \; & -{\langle p,\mathrm{div}\vec{\varphi }\rangle }_{D^{\prime }\left(\Omega \right)\times D\left(\Omega \right)}=\rho {\langle \vec{F},\;\vec{\varphi }\rangle }_{H^{-1}{\left(\Omega \right)}^{d}\times H_{0}^{1}{\left(\Omega \right)}^{d}}+\gamma \int_{\Omega }{\left|\vec{v}\right|}^{\beta -2}\vec{v}\cdot \vec{\varphi }\;dx, \end{array}$$

*for any vector function $\vec{\varphi }\in D{\left(\Omega \right)}^{d}$, and the following inequality is valid:*

(13) $$\mu \int_{\Omega }|D\vec{v}{|}^{2}\;dx\leq \rho {\langle \vec{F},\;\vec{v}\rangle }_{H^{-1}{\left(\Omega \right)}^{d}\times H_{0}^{1}{\left(\Omega \right)}^{d}}+\gamma \int_{\Omega }{\left|\vec{v}\right|}^{\beta }\;dx\;\left(the\;energy\;estimate\right).$$

**Remark** **2.** *We use the terminology “full weak solution” to emphasize that the definition of a weak solution contains a velocity–pressure pair, not just the velocity field (cf. [34], Page 234, Definition IV.1.1).*

**Theorem** **1** (Main results)**.** *Suppose that* (11) *holds. Then:*

(a) *problem *(4)* has at least one full weak solution $\left(\vec{v},p\right)\in V^{1}\left(\Omega \right)\times D^{\prime }\left(\Omega \right);$*
(b) *if $\left({\vec{v}}_{*},p_{*}\right)$ is a full weak solution of problem *(4)* and ${\vec{v}}_{*}\in H^{2}{\left(\Omega \right)}^{d}$, then*
  (14) $$\mu \int_{\Omega }|D{\vec{v}}_{*}{|}^{2}\;dx=\rho {\langle \vec{F},\;{\vec{v}}_{*}\rangle }_{H^{-1}{\left(\Omega \right)}^{d}\times H_{0}^{1}{\left(\Omega \right)}^{d}}+\gamma \int_{\Omega }{\left|{\vec{v}}_{*}\right|}^{\beta }\;dx;$$
(c) *if ${\left\{\left({\vec{v}}_{\alpha_{n}},p_{\alpha_{n}}\right)\right\}}_{n=1}^{\infty }$ is a sequence such that, for any n∈N, the pair $\left({\vec{v}}_{\alpha_{n}},p_{\alpha_{n}}\right)$ is a full weak solution of problem *(4)* with $\alpha =\alpha_{n}$ and $\lim_{n\to \infty }\alpha_{n}=0$, then one can extract a subsequence (still denoted by n) such that*
  $$\lim_{n\to \infty }{\vec{v}}_{\alpha_{n}}={\vec{v}}_{0}\;weakly\;in\;the\;space\;V^{1}{\left(\Omega \right)}^{d},$$
  *where ${\vec{v}}_{0}$ is a weak solution of the damped Navier–Stokes system* (5)*.*

The proof of this theorem is given in Section 5.

## 4. Auxiliary Results

### 4.1. Continuity of Superposition Operator (Nemytskii Operator) in Lebesgue Spaces

**Proposition** **2** (Krasnoselskii theorem)**.** *Let O be a bounded domain in space $R^{n}$. Suppose that $\omega :O\times R^{k}\to R$ is a function satisfying the following conditions:*

- *the function $\omega (\cdot ,\vec{y}):O\to R$ is measurable for any vector $\vec{y}\in R^{k}$*;
- *the function $\omega \left(\vec{x},\cdot \right):R^{k}\to R$ is continuous for almost all $\vec{x}\in O$*;
- *there exist constants ν, $\lambda_{0}$, $\lambda_{1}$, ..., $\lambda_{k}$ and a function $\omega_{0}:O\to R$ such that*
$$\begin{array}{c} \nu >0,\;\lambda_{i}\geq 1,\;i=0,\cdots ,k,\;\omega_{0}\in L^{\lambda_{0}}\left(O\right)\\ |\omega \left(\vec{x},\vec{y}\right)|\leq \nu \sum_{i=1}^{k}{\left|y_{i}\right|}^{\lambda_{i}/\lambda_{0}}+\omega_{0}\left(\vec{x}\right) \end{array}$$
*for any $\vec{y}\in R^{k}$ and almost all $\vec{x}\in O$.*

*Then the superposition operator $N_{\omega }:L^{\lambda_{1}}\left(O\right)\times \cdots \times L^{\lambda_{k}}\left(O\right)\to L^{\lambda_{0}}\left(O\right)$ defined as*

$$N_{\omega }\left[u_{1},\cdots ,u_{k}\right]\left(\vec{x}\right)\overset{\mathrm{def}}{=}\omega \left(\vec{x},u_{1}\left(\vec{x}\right),\cdots ,u_{k}\left(\vec{x}\right)\right)$$

*is a bounded continuous mapping.*

The proof of this proposition can be found in [35].

### 4.2. Solvability of Equations Involving ${\left(S\right)}_{+}$-Operators

Suppose *E* is a separable reflexive Banach space and $E^{*}$ is the dual space of *E*.

**Definition** **2** (Demicontinuous operator)**.** *We shall say that an operator $A:E\to E^{*}$ is demicontinuous if, for any sequence ${\left\{u_{k}\right\}}_{k=1}^{\infty }\subset E$, the strong convergence $u_{k}\to u_{0}$ in E implies the weak convergence $A\left(u_{k}\right)⇀A\left(u_{0}\right)$ in $E^{*}$.*

**Definition** **3** (Weak-to-strong continuous operator)**.** *We shall say that an operator $B:E\to E^{*}$ is weak-to-strong continuous if, for any sequence ${\left\{u_{k}\right\}}_{k=1}^{\infty }\subset E$, the weak convergence $u_{k}⇀u_{0}$ in E implies the strong convergence $B\left(u_{k}\right)\to B\left(u_{0}\right)$ in $E^{*}$.*

**Definition** **4** (Monotone and strongly monotone operators)**.** *An operator $M:E\to E^{*}$ is said to be monotone if*

$${\langle M\left(u\right)-M\left(v\right),u-v\rangle }_{E^{*}\times E}\geq 0,\;\forall \;u,v\in E.$$

*Moreover, if there exists a positive constant σ such that*

$${\langle M\left(u\right)-M\left(v\right),u-v\rangle }_{E^{*}\times E}\geq \sigma {∥u-v∥}_{E}^{2},\;\forall \;u,v\in E,$$

*then the operator M is said to be strongly monotone.*

Now we recall the concept of the ${\left(S\right)}_{+}$-property [27,28], which is closely related to the monotonicity and compactness properties and is widely used in the analysis of boundary value problems for nonlinear partial differential equations.

**Definition** **5** (Operator of the class ${\left(S\right)}_{+}$)**.** *An operator $A:E\to E^{*}$ is called an ${\left(S\right)}_{+}$-operator if, for any sequence ${\left\{u_{k}\right\}}_{k=1}^{\infty }\subset E$, from the two conditions:*

$$\begin{array}{c} u_{k}⇀u_{0}\;weakly\;in\;E\;as\;k\to \infty ,\\ \underset{k\to \infty }{lim sup}{\langle A\left(u_{k}\right),u_{k}-u_{0}\rangle }_{E^{*}\times E}\leq 0 \end{array}$$

*it follows that*

$$u_{k}\to u_{0}\;strongly\;in\;E\;as\;k\to \infty .$$

**Lemma** **2** (see [36])**.** *If $M:E\to E^{*}$ is strongly monotone, then M is an ${\left(S\right)}_{+}$-operator.*

**Lemma** **3** (see [36])**.** *If $A:E\to E^{*}$ is an ${\left(S\right)}_{+}$-operator, and $B:E\to E^{*}$ is a weak-to-strong continuous operator, then the sum A+B is an ${\left(S\right)}_{+}$-operator.*

**Proposition** **3** (Acute angle theorem)**.** *Let U be an open bounded set in E and 0∈U. Suppose $A:E\to E^{*}$ is a demicontinuous bounded ${\left(S\right)}_{+}$-operator such that*

$${\langle A\left(u\right)-g,u\rangle }_{E^{*}\times E}>0,\;\forall u\in \partial U,$$

*for some functional $g\in E^{*}$. Then the equation A(u)=g has at least one solution $u_{g}\in U$.*

The proof of Proposition 3 is based on the methods of topological degree theory for ${\left(S\right)}_{+}$-operators (for details, see [28], Chapter II).

### 4.3. Solvability of One-Parameter Family of Regularized Problems

Following the approach proposed by Oskolkov [6], we consider an one-parameter family of regularized problems with a small parameter $\varepsilon_{m}\overset{\mathrm{def}}{=}1/m$:

For given m∈N, find a vector function ${\vec{v}}_{m}\in V^{3}\left(\Omega \right)$ such that

(15) $$\begin{array}{cc} \; & \varepsilon_{m}\int_{\Omega }\nabla^{3}{\vec{v}}_{m}:\nabla^{3}\vec{w}\;dx+\varepsilon_{m}\int_{\Omega }\nabla^{2}{\vec{v}}_{m}\cdot \nabla^{2}\vec{w}\;dx-\rho \sum_{i=1}^{d}\int_{\Omega }v_{mi}{\vec{v}}_{m}\cdot \frac{\partial \vec{w}}{\partial x_{i}}\;dx\\ \; & +\mu \int_{\Omega }D{\vec{v}}_{m}:D\vec{w}\;dx-\alpha \sum_{i=1}^{d}\int_{\Omega }v_{mi}D{\vec{v}}_{m}:\frac{\partial D\vec{w}}{\partial x_{i}}\;dx\\ \; & =\rho {\langle \vec{F},\;\vec{w}\rangle }_{H^{-1}{\left(\Omega \right)}^{d}\times H_{0}^{1}{\left(\Omega \right)}^{d}}+\gamma \int_{\Omega }{\left|{\vec{v}}_{m}\right|}^{\beta -2}{\vec{v}}_{m}\cdot \vec{w}\;dx \end{array}$$

for any vector function $\vec{w}\in V^{3}\left(\Omega \right)$.

**Lemma** **4** (Solvability of the regularized problem)**.** *Suppose that* (11)* holds. Then problem* (15) *has at least one solution ${\vec{v}}_{m}\in V^{3}\left(\Omega \right)$ such that*

(16) $$\varepsilon_{m}∥{\vec{v}}_{m}{∥}_{V^{3}\left(\Omega \right)}^{2}+\mu ∥{\vec{v}}_{m}{∥}_{V^{1}\left(\Omega \right)}^{2}\leq \rho {\langle \vec{F},{\vec{v}}_{m}\rangle }_{H^{-1}{\left(\Omega \right)}^{d}\times H_{0}^{1}{\left(\Omega \right)}^{d}}+\gamma {∥{\vec{v}}_{m}∥}_{L^{\beta }{\left(\Omega \right)}^{d}}^{\beta }.$$

**Proof.**  We derive the proof of this lemma in eight steps.

*Step 1.* Let us consider the following operators:

$$\begin{array}{cc} \; & A_{m}:V^{3}\left(\Omega \right)\to {\left[V^{3}\left(\Omega \right)\right]}^{*},\\ \; & {\langle A_{m}\left(\vec{v}\right),\vec{w}\rangle }_{{\left[V^{3}\left(\Omega \right)\right]}^{*}\times V^{3}\left(\Omega \right)}\overset{\mathrm{def}}{=}\varepsilon_{m}\int_{\Omega }\nabla^{3}\vec{v}:\nabla^{3}\vec{w}\;dx+\varepsilon_{m}\int_{\Omega }\nabla^{2}\vec{v}\cdot \nabla^{2}\vec{w}\;dx,\\ \; & K_{1}:V^{2}\left(\Omega \right)\to {\left[V^{3}\left(\Omega \right)\right]}^{*},\;{\langle K_{1}\left(\vec{v}\right),\vec{w}\rangle }_{{\left[V^{3}\left(\Omega \right)\right]}^{*}\times V^{3}\left(\Omega \right)}\overset{\mathrm{def}}{=}-\rho \sum_{i=1}^{d}\int_{\Omega }v_{i}\vec{v}\cdot \frac{\partial \vec{w}}{\partial x_{i}}\;dx,\\ \; & K_{2}:V^{2}\left(\Omega \right)\to {\left[V^{3}\left(\Omega \right)\right]}^{*},\;{\langle K_{2}\left(\vec{v}\right),\vec{w}\rangle }_{{\left[V^{3}\left(\Omega \right)\right]}^{*}\times V^{3}\left(\Omega \right)}\overset{\mathrm{def}}{=}\mu \int_{\Omega }D\vec{v}:D\vec{w}\;dx,\\ \; & K_{3}:V^{2}\left(\Omega \right)\to {\left[V^{3}\left(\Omega \right)\right]}^{*},\;{\langle K_{3}\left(\vec{v}\right),\vec{w}\rangle }_{{\left[V^{3}\left(\Omega \right)\right]}^{*}\times V^{3}\left(\Omega \right)}\overset{\mathrm{def}}{=}-\alpha \sum_{i=1}^{d}\int_{\Omega }v_{i}D\vec{v}:\frac{\partial D\vec{w}}{\partial x_{i}}\;dx,\\ \; & K_{4}:V^{2}\left(\Omega \right)\to {\left[V^{3}\left(\Omega \right)\right]}^{*},\;{\langle K_{4}\left(\vec{v}\right),\vec{w}\rangle }_{{\left[V^{3}\left(\Omega \right)\right]}^{*}\times V^{3}\left(\Omega \right)}\overset{\mathrm{def}}{=}-\gamma \int_{\Omega }{\left|\vec{v}\right|}^{\beta -2}\vec{v}\cdot \vec{w}\;dx,\\ \; & K:V^{2}\left(\Omega \right)\to {\left[V^{3}\left(\Omega \right)\right]}^{*},\;K\overset{\mathrm{def}}{=}\sum_{i=1}^{4}K_{i},\\ \; & I:V^{3}\left(\Omega \right)\to V^{2}\left(\Omega \right),\;I\left(\vec{v}\right)\overset{\mathrm{def}}{=}\vec{v} \end{array}$$

and rewrite problem (15) in the operator form:

(17) $$A_{m}\left({\vec{v}}_{m}\right)+K\circ I\left({\vec{v}}_{m}\right)=f$$

with the functional $f\in {\left[V^{3}\left(\Omega \right)\right]}^{*}$ that is defined by the formula

$${\langle f,\vec{w}\rangle }_{{\left[V^{3}\left(\Omega \right)\right]}^{*}\times V^{3}\left(\Omega \right)}\overset{\mathrm{def}}{=}\rho {\langle \vec{F},\;\vec{w}\rangle }_{H^{-1}{\left(\Omega \right)}^{d}\times H_{0}^{1}{\left(\Omega \right)}^{d}}.$$

*Step 2.* Clearly, the operator $A_{m}$ is continuous, and

$$\begin{array}{cc} \; & {\langle A_{m}\left(\vec{v}\right)-A_{m}\left(\vec{u}\right),\vec{v}-\vec{u}\rangle }_{{\left[V^{3}\left(\Omega \right)\right]}^{*}\times V^{3}\left(\Omega \right)}\\ \; & =\varepsilon_{m}\int_{\Omega }|\nabla^{3}\left(\vec{v}-\vec{u}\right){|}^{2}\;dx+\varepsilon_{m}\int_{\Omega }{\left|\nabla^{2}\left(\vec{v}-\vec{u}\right)\right|}^{2}\;dx\\ \; & =\varepsilon_{m}{∥\vec{v}-\vec{u}∥}_{V^{3}\left(\Omega \right)}^{2},\;\forall \;\vec{v},\vec{u}\in V^{3}\left(\Omega \right). \end{array}$$

Therefore, $A_{m}$ is strongly monotone. Moreover, in view of Lemma 2, this operator belongs to the class ${\left(S\right)}_{+}$.

*Step 3.* We claim that the operator I is weak-to-strong continuous. Indeed, this follows from the compactness of the imbedding $V^{3}\left(\Omega \right)$ into $V^{2}\left(\Omega \right)$ (see Remark 1).

*Step 4.* Let us prove the operator K is continuous. To begin with, we consider the first term $K_{1}$. Let ${\left\{{\vec{u}}_{n}\right\}}_{n=1}^{\infty }$ be an arbitrary sequence from the space $V^{2}\left(\Omega \right)$ such that

$${\vec{u}}_{n}\to {\vec{u}}_{0}\;\mathrm{strongly}\;\mathrm{in}\;V^{2}\left(\Omega \right)\;\mathrm{as}\;n\to \infty .$$

We must show that

(18) $$K_{1}\left({\vec{u}}_{n}\right)\to K_{1}\left({\vec{u}}_{0}\right)\;\mathrm{strongly}\;\mathrm{in}\;{\left[V^{3}\left(\Omega \right)\right]}^{*}\;\mathrm{as}\;n\to \infty .$$

In view of (7), we have

(19) $${\vec{u}}_{n}\to {\vec{u}}_{0}\;\mathrm{strongly}\;\mathrm{in}\;C{\left(\bar{\Omega }\right)}^{d}\;\mathrm{as}\;n\to \infty .$$

Let $\vec{w}$ be an arbitrary vector function from the space $V^{3}\left(\Omega \right)$. We observe that

$$\begin{array}{cc} \; & {\langle K_{1}\left({\vec{u}}_{n}\right)-K_{1}\left({\vec{u}}_{0}\right),\vec{w}\rangle }_{{\left[V^{3}\left(\Omega \right)\right]}^{*}\times V^{3}\left(\Omega \right)}\\ \; & =-\rho \sum_{i=1}^{d}\int_{\Omega }u_{ni}{\vec{u}}_{n}\cdot \frac{\partial \vec{w}}{\partial x_{i}}\;dx+\rho \sum_{i=1}^{d}\int_{\Omega }u_{0i}{\vec{u}}_{0}\cdot \frac{\partial \vec{w}}{\partial x_{i}}\;dx\\ \; & =\rho \sum_{i=1}^{d}\int_{\Omega }\left(u_{0i}-u_{ni}\right){\vec{u}}_{n}\cdot \frac{\partial \vec{w}}{\partial x_{i}}\;dx+\rho \sum_{i=1}^{d}\int_{\Omega }u_{0i}\left({\vec{u}}_{0}-{\vec{u}}_{n}\right)\cdot \frac{\partial \vec{w}}{\partial x_{i}}\;dx. \end{array}$$

An application of Hölder’s inequality then implies that

$$\begin{array}{cc} \; & |{\langle K_{1}\left({\vec{u}}_{n}\right)-K_{1}\left({\vec{u}}_{0}\right),\vec{w}\rangle }_{{\left[V^{3}\left(\Omega \right)\right]}^{*}\times V^{3}\left(\Omega \right)}|\\ \; & \leq C\rho ∥{\vec{u}}_{n}-{\vec{u}}_{0}{∥}_{L^{4}{\left(\Omega \right)}^{d}}\underset{k\in N\cup \{0\}}{\sup}∥{\vec{u}}_{k}{∥}_{L^{4}{\left(\Omega \right)}^{d}}{∥\vec{w}∥}_{V^{3}\left(\Omega \right)}, \end{array}$$

with a constant *C* independent of *n*. Hence, we have

$$∥K_{1}\left({\vec{u}}_{n}\right)-K_{1}\left({\vec{u}}_{0}\right){∥}_{{\left[V^{3}\left(\Omega \right)\right]}^{*}}\leq C\rho ∥{\vec{u}}_{n}-{\vec{u}}_{0}{∥}_{L^{4}{\left(\Omega \right)}^{d}}\underset{k\in N\cup \{0\}}{\sup}{∥{\vec{u}}_{k}∥}_{L^{4}{\left(\Omega \right)}^{d}}.$$

Taking into account (19), we pass to the limit n→∞ in the last inequality and obtain

$$\lim_{n\to \infty }{∥K_{1}\left({\vec{u}}_{n}\right)-K_{1}\left({\vec{u}}_{0}\right)∥}_{{\left[V^{3}\left(\Omega \right)\right]}^{*}}=0.$$

This means that (18) holds.

By similar arguments, one can show the continuity of the operators $K_{2}$, $K_{3}$, and $K_{4}$. Thus, the sum $K=\sum_{i=1}^{4}K_{i}$ is continuous, while the operator K∘I is weak-to-strong continuous.

*Step 5.* Applying Lemma 3, we deduce that $A_{m}+K\circ I$ is an ${\left(S\right)}_{+}$-operator.

*Step 6.* Note that

(20) $$\begin{array}{cc} {\langle A_{m}\left(\vec{v}\right),\vec{v}\rangle }_{{\left[V^{3}\left(\Omega \right)\right]}^{*}\times V^{3}\left(\Omega \right)}\; & =\varepsilon_{m}{∥\vec{v}∥}_{V^{3}\left(\Omega \right)}^{2}, \end{array}$$

(21) $$\begin{array}{cc} {\langle K_{2}\circ I\left(\vec{v}\right),\vec{v}\rangle }_{{\left[V^{3}\left(\Omega \right)\right]}^{*}\times V^{3}\left(\Omega \right)}\; & =\mu ∥\vec{v}{∥}_{V^{1}\left(\Omega \right)}^{2}, \end{array}$$

(22) $$\begin{array}{cc} {\langle K_{4}\circ I\left(\vec{v}\right),\vec{v}\rangle }_{{\left[V^{3}\left(\Omega \right)\right]}^{*}\times V^{3}\left(\Omega \right)}\; & =-\gamma ∥\vec{v}{∥}_{L^{\beta }{\left(\Omega \right)}^{d}}^{\beta }, \end{array}$$

for any $\vec{v}\in V^{3}\left(\Omega \right)$. Moreover, integrating by parts, we obtain

(23) $$\begin{array}{cc} {\langle K_{1}\circ I\left(\vec{v}\right),\vec{v}\rangle }_{{\left[V^{3}\left(\Omega \right)\right]}^{*}\times V^{3}\left(\Omega \right)}= & \frac{\rho }{2}\sum_{i=1}^{d}\int_{\Omega }v_{i}\frac{\partial |\vec{v}{|}^{2}}{\partial x_{i}}\;dx\\ = & \frac{\rho }{2}\int_{\partial \Omega }\underset{=0}{\underset{︸}{\left(\vec{v}\cdot \vec{n}\right){\left|\vec{v}\right|}^{2}}}\;dx-\frac{\rho }{2}\int_{\Omega }\underset{=0}{\underset{︸}{\left(\mathrm{div}\;\vec{v}\right)}}{\left|\vec{v}\right|}^{2}\;dx\\ = & 0 \end{array}$$

and

(24) $$\begin{array}{cc} {\langle K_{3}\circ I\left(\vec{v}\right),\vec{v}\rangle }_{{\left[V^{3}\left(\Omega \right)\right]}^{*}\times V^{3}\left(\Omega \right)}= & \frac{\alpha }{2}\sum_{i=1}^{d}\int_{\Omega }v_{i}\frac{\partial |D\vec{v}{|}^{2}}{\partial x_{i}}\;dx\\ = & \frac{\alpha }{2}\int_{\partial \Omega }\underset{=0}{\underset{︸}{\left(\vec{v}\cdot \vec{n}\right)}}|D\vec{v}{|}^{2}\;dx-\frac{\alpha }{2}\int_{\Omega }\underset{=0}{\underset{︸}{\left(\mathrm{div}\;\vec{v}\right)}}{\left|D\vec{v}\right|}^{2}\;dx\\ = & 0, \end{array}$$

where $\vec{n}=\vec{n}\left(\vec{x}\right)$ is the unit outward normal vector to the surface ∂Ω.

From (20)–(24) it follows that

(25) $$\begin{array}{cc} \; & {\langle A_{m}\left(\vec{v}\right)+K\circ I\left(\vec{v}\right)-f,\vec{v}\rangle }_{{\left[V^{3}\left(\Omega \right)\right]}^{*}\times V^{3}\left(\Omega \right)}\\ \; & =\varepsilon_{m}∥\vec{v}{∥}_{V^{3}\left(\Omega \right)}^{2}+\mu ∥\vec{v}{∥}_{V^{1}\left(\Omega \right)}^{2}-\gamma {∥\vec{v}∥}_{L^{\beta }{\left(\Omega \right)}^{d}}^{\beta }-\rho {\langle \vec{F},\;\vec{w}\rangle }_{H^{-1}{\left(\Omega \right)}^{d}\times H_{0}^{1}{\left(\Omega \right)}^{d}}. \end{array}$$

Taking into account the relations:

$$∥\vec{v}{∥}_{L^{\beta }{\left(\Omega \right)}^{d}}\leq {∥\mathrm{Id}∥}_{L(V^{3}\left(\Omega \right),L^{\beta }{\left(\Omega \right)}^{d})}{∥\vec{v}∥}_{V^{3}\left(\Omega \right)}$$

and

$$\begin{array}{cc} |{\langle \vec{F},\;\vec{w}\rangle }_{H^{-1}{\left(\Omega \right)}^{d}\times H_{0}^{1}{\left(\Omega \right)}^{d}}|\leq & ∥\vec{F}{∥}_{H^{-1}{\left(\Omega \right)}^{d}}{∥\vec{w}∥}_{H_{0}^{1}{\left(\Omega \right)}^{d}}\\ \leq & ∥\vec{F}{∥}_{H^{-1}{\left(\Omega \right)}^{d}}{∥\mathrm{Id}∥}_{L(V^{3}\left(\Omega \right),H_{0}^{1}{\left(\Omega \right)}^{d})}{∥\vec{v}∥}_{V^{3}\left(\Omega \right)}, \end{array}$$

we derive from (25) the estimate

(26) $$\begin{array}{cc} \; & {\langle A_{m}\left(\vec{v}\right)+K\circ I\left(\vec{v}\right)-f,\vec{v}\rangle }_{{\left[V^{3}\left(\Omega \right)\right]}^{*}\times V^{3}\left(\Omega \right)}\\ \; & \geq \varepsilon_{m}∥\vec{v}{∥}_{V^{3}\left(\Omega \right)}^{2}+\mu ∥\vec{v}{∥}_{V^{1}\left(\Omega \right)}^{2}-\gamma 1_{\left[0,+\infty \right)}{\left(\gamma \right)∥\mathrm{Id}∥}_{L(V^{3}\left(\Omega \right),L^{\beta }{\left(\Omega \right)}^{d})}^{\beta }{∥\vec{v}∥}_{V^{3}\left(\Omega \right)}^{\beta }\\ \; & -\rho ∥\vec{F}{∥}_{H^{-1}{\left(\Omega \right)}^{d}}{∥\mathrm{Id}∥}_{L(V^{3}\left(\Omega \right),H_{0}^{1}{\left(\Omega \right)}^{d})}{∥\vec{v}∥}_{V^{3}\left(\Omega \right)}, \end{array}$$

where $1_{\left[0,+\infty \right)}$ is the characteristic (indicator) function of the set [0,+∞) as a subset of R.

Let us introduce the function $\Phi \left[\Omega ,m,\gamma ,\beta ,\rho ,\vec{F}\right]:\left[0,+\infty \right)\to R$ by the formula

$$\begin{array}{cc} \Phi \left[\Omega ,m,\gamma ,\beta ,\rho ,\vec{F}\right]\left(r\right)\overset{\mathrm{def}}{=}\; & \frac{1}{m}r^{2}-\gamma 1_{\left[0,\infty \right)}\left(\gamma \right){∥\mathrm{Id}∥}_{L(V^{3}\left(\Omega \right),L^{\beta }{\left(\Omega \right)}^{d})}^{\beta }r^{\beta }\\ \; & -\rho ∥\vec{F}{∥}_{H^{-1}{\left(\Omega \right)}^{d}}{∥\mathrm{Id}∥}_{L(V^{3}\left(\Omega \right),H_{0}^{1}{\left(\Omega \right)}^{d})}r \end{array}$$

and rewrite (26) as

$$\begin{array}{cc} \; & {\langle A_{m}\left(\vec{v}\right)+K\circ I\left(\vec{v}\right)-f,\vec{v}\rangle }_{{\left[V^{3}\left(\Omega \right)\right]}^{*}\times V^{3}\left(\Omega \right)}\\ \; & \geq \mu ∥\vec{v}{∥}_{V^{1}\left(\Omega \right)}^{2}+\Phi \left[\Omega ,m,\gamma ,\beta ,\rho ,\vec{F}\right](∥\vec{v}{∥}_{V^{3}\left(\Omega \right)}). \end{array}$$

Since β∈B(d,γ), we see that

$$\lim_{r\to +\infty }\Phi \left[\Omega ,m,\gamma ,\beta ,\rho ,\vec{F}\right]\left(r\right)=+\infty .$$

Hence, there exists a positive number $R_{0}\left[\Omega ,m,\gamma ,\beta ,\rho ,\vec{F}\right]$ such that

$${\langle A_{m}\left(\vec{v}\right)+K\circ I\left(\vec{v}\right)-f,\vec{v}\rangle }_{{\left[V^{3}\left(\Omega \right)\right]}^{*}\times V^{3}\left(\Omega \right)}>0,$$

for any vector function $\vec{v}\in V^{3}\left(\Omega \right)$ satisfying the inequality

$$∥\vec{v}{∥}_{V^{3}\left(\Omega \right)}\geq R_{0}\left[\Omega ,m,\gamma ,\beta ,\rho ,\vec{F}\right].$$

*Step 7.* Let

$$B_{R_{0}\left[\Omega ,m,\gamma ,\beta ,\rho ,\vec{F}\right]}\left(\vec{0}\right)\overset{\mathrm{def}}{=}\left\{\vec{w}\in V^{3}\left(\Omega \right):\;{∥\vec{w}∥}_{V^{3}\left(\Omega \right)}<R_{0}\left[\Omega ,m,\gamma ,\beta ,\rho ,\vec{F}\right]\right\}.$$

Applying Proposition 3 to (17), we conclude that, for any m∈N, problem (15) has a solution ${\vec{v}}_{m}$ in the ball $B_{R_{0}\left[\Omega ,m,\gamma ,\beta ,\rho ,\vec{F}\right]}\left(\vec{0}\right)$.

*Step 8.* Estimate (16) is obtained by substituting $\vec{w}={\vec{v}}_{m}$ into (15) and taking into account relations (20)–(24) with $\vec{v}={\vec{v}}_{m}$. Thus, Lemma 4 is proved. □

## 5. Proof of Main Results

First we establish the existence result (a).

In view of Lemma 4, regularized problem (15) is solvable for any m∈N. Consider a sequence ${\left\{{\vec{v}}_{m}\right\}}_{m=1}^{\infty }$ such that the vector function ${\vec{v}}_{m}\in V^{3}\left(\Omega \right)$ is a solution of (15) satisfying inequality (16).

Let us show that the sequence ${\left\{{\vec{v}}_{m}\right\}}_{m=1}^{\infty }$ is bounded in the space $V^{1}\left(\Omega \right)$. From (16) it follows that

(27) $$\mu ∥{\vec{v}}_{m}{∥}_{V^{1}\left(\Omega \right)}^{2}\leq \rho {\langle \vec{F},{\vec{v}}_{m}\rangle }_{H^{-1}{\left(\Omega \right)}^{d}\times H_{0}^{1}{\left(\Omega \right)}^{d}}+\gamma {∥{\vec{v}}_{m}∥}_{L^{\beta }{\left(\Omega \right)}^{d}}^{\beta },$$

for any m∈N. Moreover, we have

(28) $$\begin{array}{cc} {\langle \vec{F},{\vec{v}}_{m}\rangle }_{H^{-1}{\left(\Omega \right)}^{d}\times H_{0}^{1}{\left(\Omega \right)}^{d}}\leq & ∥\vec{F}{∥}_{H^{-1}{\left(\Omega \right)}^{d}}{∥{\vec{v}}_{m}∥}_{H_{0}^{1}{\left(\Omega \right)}^{d}}\\ \leq & ∥\vec{F}{∥}_{H^{-1}{\left(\Omega \right)}^{d}}{∥\mathrm{Id}∥}_{L(V^{1}\left(\Omega \right),H_{0}^{1}{\left(\Omega \right)}^{d})}{∥{\vec{v}}_{m}∥}_{V^{1}\left(\Omega \right)}. \end{array}$$

Combining (27) and (28), we obtain

(29) $$\mu ∥{\vec{v}}_{m}{∥}_{V^{1}\left(\Omega \right)}^{2}\leq \rho ∥\vec{F}{∥}_{H^{-1}{\left(\Omega \right)}^{d}}{∥\mathrm{Id}∥}_{L(V^{1}\left(\Omega \right),H_{0}^{1}{\left(\Omega \right)}^{d})}∥{\vec{v}}_{m}{∥}_{V^{1}\left(\Omega \right)}+\gamma {∥{\vec{v}}_{m}∥}_{L^{\beta }{\left(\Omega \right)}^{d}}^{\beta }.$$

If γ≤0, then we obviously have

$$\mu ∥{\vec{v}}_{m}{∥}_{V^{1}\left(\Omega \right)}^{2}\leq \rho ∥\vec{F}{∥}_{H^{-1}{\left(\Omega \right)}^{d}}{∥\mathrm{Id}∥}_{L(V^{1}\left(\Omega \right),H_{0}^{1}{\left(\Omega \right)}^{d})}{∥{\vec{v}}_{m}∥}_{V^{1}\left(\Omega \right)},$$

whence

(30) $$∥{\vec{v}}_{m}{∥}_{V^{1}\left(\Omega \right)}\leq \frac{\rho ∥\vec{F}{∥}_{H^{-1}{\left(\Omega \right)}^{d}}{∥\mathrm{Id}∥}_{L(V^{1}\left(\Omega \right),H_{0}^{1}{\left(\Omega \right)}^{d})}}{\mu }.$$

Now, consider the case when γ>0. We rewrite (29) as follows:

(31) $$\mu ∥{\vec{v}}_{m}{∥}_{V^{1}\left(\Omega \right)}^{2}-\gamma ∥{\vec{v}}_{m}{∥}_{L^{\beta }{\left(\Omega \right)}^{d}}^{\beta }\leq \rho ∥\vec{F}{∥}_{H^{-1}{\left(\Omega \right)}^{d}}{∥\mathrm{Id}∥}_{L(V^{1}\left(\Omega \right),H_{0}^{1}{\left(\Omega \right)}^{d})}{∥{\vec{v}}_{m}∥}_{V^{1}\left(\Omega \right)}.$$

Noticing that

$$∥{\vec{v}}_{m}{∥}_{L^{\beta }{\left(\Omega \right)}^{d}}^{\beta }\leq {∥\mathrm{Id}∥}_{L(V^{1}\left(\Omega \right),L^{\beta }{\left(\Omega \right)}^{d})}^{\beta }{∥{\vec{v}}_{m}∥}_{V^{1}\left(\Omega \right)}^{\beta },$$

one can derive from (31) the inequality

$$\begin{array}{cc} \; & \mu ∥{\vec{v}}_{m}{∥}_{V^{1}\left(\Omega \right)}^{2}-{\gamma ∥\mathrm{Id}∥}_{L(V^{1}\left(\Omega \right),L^{\beta }{\left(\Omega \right)}^{d})}^{\beta }{∥{\vec{v}}_{m}∥}_{V^{1}\left(\Omega \right)}^{\beta }\\ \; & \leq \rho ∥\vec{F}{∥}_{H^{-1}{\left(\Omega \right)}^{d}}{∥\mathrm{Id}∥}_{L(V^{1}\left(\Omega \right),H_{0}^{1}{\left(\Omega \right)}^{d})}{∥{\vec{v}}_{m}∥}_{V^{1}\left(\Omega \right)}, \end{array}$$

whence

(32) $$\mu ∥{\vec{v}}_{m}{∥}_{V^{1}\left(\Omega \right)}-{\gamma ∥\mathrm{Id}∥}_{L(V^{1}\left(\Omega \right),L^{\beta }{\left(\Omega \right)}^{d})}^{\beta }∥{\vec{v}}_{m}{∥}_{V^{1}\left(\Omega \right)}^{\beta -1}\leq \rho ∥\vec{F}{∥}_{H^{-1}{\left(\Omega \right)}^{d}}{∥\mathrm{Id}∥}_{L(V^{1}\left(\Omega \right),H_{0}^{1}{\left(\Omega \right)}^{d})}.$$

Let us introduce the function Ψ[Ω,μ,γ,β]:[0,+∞)→R by the formula

$$\Psi \left[\Omega ,\mu ,\gamma ,\beta \right]\left(r\right)\overset{\mathrm{def}}{=}\mu r-\gamma {∥\mathrm{Id}∥}_{L(V^{1}\left(\Omega \right),L^{\beta }{\left(\Omega \right)}^{d})}^{\beta }r^{\beta -1}$$

and rewrite (32) in the form

(33) $$\Psi \left[\Omega ,\mu ,\gamma ,\beta \right](∥{\vec{v}}_{m}{∥}_{V^{1}\left(\Omega \right)})\leq \rho ∥\vec{F}{∥}_{H^{-1}{\left(\Omega \right)}^{d}}{∥\mathrm{Id}∥}_{L(V^{1}\left(\Omega \right),H_{0}^{1}{\left(\Omega \right)}^{d})}.$$

Since β∈B(d,γ), we see that β∈(0,1). Therefore,

$$\lim_{r\to +\infty }\Psi \left[\Omega ,\mu ,\gamma ,\beta \right]\left(r\right)=+\infty ,$$

and there exists a positive number $R_{1}\left[\Omega ,\mu ,\gamma ,\beta ,\rho ,\vec{F}\right]$ such that

$$\Psi \left[\Omega ,\mu ,\gamma ,\beta \right]\left(r\right)>\rho ∥\vec{F}{∥}_{H^{-1}{\left(\Omega \right)}^{d}}{∥\mathrm{Id}∥}_{L(V^{1}\left(\Omega \right),H_{0}^{1}{\left(\Omega \right)}^{d})},$$

for any *r* satisfying the following inequality

$$r\geq R_{1}\left[\Omega ,\mu ,\gamma ,\beta ,\rho ,\vec{F}\right].$$

Then, in view of (33), we arrive at the estimate

(34) $$∥{\vec{v}}_{m}{∥}_{V^{1}\left(\Omega \right)}\leq R_{1}\left[\Omega ,\mu ,\gamma ,\beta ,\rho ,\vec{F}\right].$$

Taking into account inequalities (30) and (34), we deduce that, in both cases: γ≤0 and γ>0, the sequence ${\left\{{\vec{v}}_{m}\right\}}_{m=1}^{\infty }$ is bounded in the space $V^{1}\left(\Omega \right)$. Therefore, without loss of generality it can be assumed that

(35) $$\begin{array}{cc} \; & {\vec{v}}_{m}⇀\vec{v}\;\;\mathrm{weakly}\;\mathrm{in}\;V^{1}\left(\Omega \right)\;\mathrm{as}\;m\to \infty , \end{array}$$

(36) $$\begin{array}{cc} \; & {\vec{v}}_{m}\to \vec{v}\;\;\mathrm{strongly}\;\mathrm{in}\;L^{s}{\left(\Omega \right)}^{d},\;s\in S\left(d\right),\;\mathrm{as}\;m\to \infty , \end{array}$$

for some vector function $\vec{v}$ from the space $V^{1}\left(\Omega \right)$.

Moreover, using (6), (35), and the inclusion β∈B(d,γ), we obtain

(37) $${\vec{v}}_{m}\to \vec{v}\;\mathrm{strongly}\;\mathrm{in}\;L^{2\beta -1}{\left(\Omega \right)}^{d}\;\mathrm{as}\;m\to \infty .$$

Let us show that

(38) $$|{\vec{v}}_{m}{|}^{\beta -2}{\vec{v}}_{m}\to {\left|\vec{v}\right|}^{\beta -2}\vec{v}\;\mathrm{strongly}\;\mathrm{in}\;L^{2}{\left(\Omega \right)}^{d}asm\to \infty .$$

Consider the real-valued functions $h_{i}$, i=1,⋯,d, defined as follows:

$$h_{i}:R^{d}\to R,\;h_{i}\left(\vec{y}\right)\overset{\mathrm{def}}{=}{\left|\vec{y}\right|}^{\beta -2}y_{i}.$$

Using the following inequality

$${\left|\sum_{j=1}^{d}a_{j}\right|}^{q}\leq 2^{(d-1)q}\sum_{j=1}^{d}{\left|a_{j}\right|}^{q},\;\forall \;a_{1},\cdots ,a_{d}\in R,\;q>0,\;d=2,3,$$

we derive

(39) $$\begin{array}{cc} |h_{i}\left(\vec{y}\right)|= & {\left(\sum_{j=1}^{d}y_{j}^{2}\right)}^{(\beta -2)/2}\left|y_{i}\right|\\ \leq & {\left(\sum_{j=1}^{d}y_{j}^{2}\right)}^{(\beta -1)/2}\\ \leq & 2^{(d-1)(\beta -1)/2}\sum_{j=1}^{d}{\left|y_{j}\right|}^{\beta -1},\;\forall \;i=1,\cdots ,d. \end{array}$$

Moreover, by applying Young’s inequality, we obtain

(40) $$|y_{i}{|}^{\beta -1}\leq \frac{2\beta -2}{2\beta -1}{\left|y_{i}\right|}^{(2\beta -1)/2}+\frac{1}{2\beta -1},\;\forall \;i=1,\cdots ,d.$$

Combining (39) and (40), we arrive at the estimate

(41) $$|h_{i}\left(\vec{y}\right)|\leq \zeta_{1}\left(d,\beta \right)\sum_{j=1}^{d}{\left|y_{j}\right|}^{(2\beta -1)/2}+\zeta_{2}\left(d,\beta \right),\;\forall \;i=1,\cdots ,d,$$

with

$$\begin{array}{cc} \zeta_{1}\left(d,\beta \right) & \overset{\mathrm{def}}{=}\frac{2^{(d-1)(\beta -1)/2}\left(2\beta -2\right)}{2\beta -1},\\ \zeta_{2}\left(d,\beta \right) & \overset{\mathrm{def}}{=}\frac{2^{(d-1)(\beta -1)/2}d}{2\beta -1}. \end{array}$$

Let us consider the operator H given by

$$H:L^{2\beta -1}{\left(\Omega \right)}^{d}\to L^{2}{\left(\Omega \right)}^{d},\;H\left(\vec{v}\right)\overset{\mathrm{def}}{=}\left(h_{1}\left(\vec{v}\right),\cdots ,h_{d}\left(\vec{v}\right)\right).$$

Using Proposition 2 and estimate (41), we deduce that the operator H is well defined and continuous. Therefore, from (37) it follows that

$$H\left({\vec{v}}_{m}\right)\to H\left(\vec{v}\right)\;\mathrm{strongly}\;\mathrm{in}\;L^{2}{\left(\Omega \right)}^{d}\;\mathrm{as}\;m\to \infty ,$$

and hence, (38) holds.

Let $\vec{\psi }$ be an arbitrary vector function from the set $D_{\mathrm{sol}}{\left(\Omega \right)}^{d}$. Since ${\vec{v}}_{m}$ is a solution to (15), we have

$$\begin{array}{cc} \; & \varepsilon_{m}\int_{\Omega }\nabla^{3}{\vec{v}}_{m}:\nabla^{3}\vec{\psi }\;dx+\varepsilon_{m}\int_{\Omega }\nabla^{2}{\vec{v}}_{m}\cdot \nabla^{2}\vec{\psi }\;dx-\rho \sum_{i=1}^{d}\int_{\Omega }v_{mi}{\vec{v}}_{m}\cdot \frac{\partial \vec{\psi }}{\partial x_{i}}\;dx\\ \; & +\mu \int_{\Omega }D{\vec{v}}_{m}:D\vec{\psi }\;dx-\alpha \sum_{i=1}^{d}\int_{\Omega }v_{mi}D{\vec{v}}_{m}:\frac{\partial D\vec{\psi }}{\partial x_{i}}\;dx\\ \; & =\rho {\langle \vec{F},\;\vec{\psi }\rangle }_{H^{-1}{\left(\Omega \right)}^{d}\times H_{0}^{1}{\left(\Omega \right)}^{d}}+\gamma \int_{\Omega }{\left|{\vec{v}}_{m}\right|}^{\beta -2}{\vec{v}}_{m}\cdot \vec{\psi }\;dx. \end{array}$$

After integration by parts in the first and second terms in the left-hand side of the last equality, we obtain

(42) $$\begin{array}{cc} \; & -\varepsilon_{m}\int_{\Omega }{\vec{v}}_{m}\cdot \nabla^{6}\vec{\psi }\;dx+\varepsilon_{m}\int_{\Omega }{\vec{v}}_{m}\cdot \nabla^{4}\vec{\psi }\;dx-\rho \sum_{i=1}^{d}\int_{\Omega }v_{mi}{\vec{v}}_{m}\cdot \frac{\partial \vec{\psi }}{\partial x_{i}}\;dx\\ \; & +\mu \int_{\Omega }D{\vec{v}}_{m}:D\vec{\psi }\;dx-\alpha \sum_{i=1}^{d}\int_{\Omega }v_{mi}D{\vec{v}}_{m}:\frac{\partial D\vec{\psi }}{\partial x_{i}}\;dx\\ \; & =\rho {\langle \vec{F},\;\vec{\psi }\rangle }_{H^{-1}{\left(\Omega \right)}^{d}\times H_{0}^{1}{\left(\Omega \right)}^{d}}+\gamma \int_{\Omega }{\left|{\vec{v}}_{m}\right|}^{\beta -2}{\vec{v}}_{m}\cdot \vec{\psi }\;dx. \end{array}$$

Taking into account (35)–(38), we pass to the limit m→∞ in equality (42); this gives

(43) $$\begin{array}{cc} \; & -\rho \sum_{i=1}^{d}\int_{\Omega }v_{i}\vec{v}\cdot \frac{\partial \vec{\psi }}{\partial x_{i}}\;dx+\mu \int_{\Omega }D\vec{v}:D\vec{\psi }\;dx-\alpha \sum_{i=1}^{d}\int_{\Omega }v_{i}D\vec{v}:\frac{\partial D\vec{\psi }}{\partial x_{i}}\;dx\\ \; & =\rho {\langle \vec{F},\;\vec{\psi }\rangle }_{H^{-1}{\left(\Omega \right)}^{d}\times H_{0}^{1}{\left(\Omega \right)}^{d}}+\gamma \int_{\Omega }{\left|\vec{v}\right|}^{\beta -2}\vec{v}\cdot \vec{\psi }\;dx. \end{array}$$

Using the De Rham theorem (see, e. g., [32], Chapter I, Section 1.4, Proposition 1.1) and equality (43), we deduce that there exists $p\in D^{\prime }\left(\Omega \right)$ such that the pair $\left(\vec{v},p\right)$ satisfies the first equality of system (4) in the distributions sense. This implies equality (12).

Further, we substitute ${\vec{v}}_{m}$ for $\vec{w}$ in equality (15). Since the third and fifth terms in the left-side of the resulting equality are equal to zero, we see that

(44) $$\mu \int_{\Omega }|D{\vec{v}}_{m}{|}^{2}\;dx\leq \rho {\langle \vec{F},{\vec{v}}_{m}\rangle }_{H^{-1}{\left(\Omega \right)}^{d}\times H_{0}^{1}{\left(\Omega \right)}^{d}}+\gamma \int_{\Omega }{\left|{\vec{v}}_{m}\right|}^{\beta }\;dx.$$

From the imbedding $L^{2\beta -1}\left(\Omega \right)↪L^{\beta }\left(\Omega \right)$ and (37) it follows that

$${\vec{v}}_{m}\to \vec{v}\;\mathrm{strongly}\;\mathrm{in}\;L^{\beta }{\left(\Omega \right)}^{d}asm\to \infty ,$$

whence

(45) $$\int_{\Omega }|\vec{v}{|}^{\beta }\;dx=\lim_{m\to \infty }\int_{\Omega }{\left|{\vec{v}}_{m}\right|}^{\beta }\;dx.$$

Moreover, taking into account (35), we obtain

(46) $$\begin{array}{cc} \int_{\Omega }{\left|D\vec{v}\right|}^{2}\;dx\; & \leq \underset{m\to \infty }{lim inf}\int_{\Omega }{\left|D{\vec{v}}_{m}\right|}^{2}\;dx, \end{array}$$

(47) $$\begin{array}{cc} {\langle \vec{F},\vec{v}\rangle }_{H^{-1}{\left(\Omega \right)}^{d}\times H_{0}^{1}{\left(\Omega \right)}^{d}}\; & =\lim_{m\to \infty }{\langle \vec{F},{\vec{v}}_{m}\rangle }_{H^{-1}{\left(\Omega \right)}^{d}\times H_{0}^{1}{\left(\Omega \right)}^{d}}. \end{array}$$

Using (45)–(47), we pass to the inferior limit in both sides of inequality (44) and arrive at estimate (13). Thus, we have established that the pair $\left(\vec{v},p\right)$ is a full weak solution of problem (4).

Now, we prove the statement (b). Substituting ${\vec{v}}_{*}$ for $\vec{v}$ and $\vec{\psi }$ for $\vec{\varphi }$ in (12), we get

(48) $$\begin{array}{cc} \; & -\rho \sum_{i=1}^{d}\int_{\Omega }v_{*i}{\vec{v}}_{*}\cdot \frac{\partial \vec{\psi }}{\partial x_{i}}\;dx+\mu \int_{\Omega }D{\vec{v}}_{*}:D\vec{\psi }\;dx-\alpha \sum_{i=1}^{d}\int_{\Omega }v_{*i}D{\vec{v}}_{*}:\frac{\partial D\vec{\psi }}{\partial x_{i}}\;dx\\ \; & =\rho {\langle \vec{F},\;\vec{\psi }\rangle }_{H^{-1}{\left(\Omega \right)}^{d}\times H_{0}^{1}{\left(\Omega \right)}^{d}}+\gamma \int_{\Omega }{\left|{\vec{v}}_{*}\right|}^{\beta -2}{\vec{v}}_{*}\cdot \vec{\psi }\;dx,\;\forall \;\vec{\psi }\in D_{\mathrm{sol}}{\left(\Omega \right)}^{d}. \end{array}$$

Since the set $D_{\mathrm{sol}}{\left(\Omega \right)}^{d}$ is dense in the space $V^{2}\left(\Omega \right)$ and ${\vec{v}}_{*}\in H^{2}{\left(\Omega \right)}^{d}$, equality (48) remains valid if we replace $\vec{\psi }$ by an arbitrary vector function $\vec{\eta }$ from the space $V^{2}\left(\Omega \right)$:

$$\begin{array}{cc} \; & -\rho \sum_{i=1}^{d}\int_{\Omega }v_{*i}{\vec{v}}_{*}\cdot \frac{\partial \vec{\eta }}{\partial x_{i}}\;dx+\mu \int_{\Omega }D{\vec{v}}_{*}:D\vec{\eta }\;dx-\alpha \sum_{i=1}^{d}\int_{\Omega }v_{*i}D{\vec{v}}_{*}:\frac{\partial D\vec{\eta }}{\partial x_{i}}\;dx\\ \; & =\rho {\langle \vec{F},\;\vec{\eta }\rangle }_{H^{-1}{\left(\Omega \right)}^{d}\times H_{0}^{1}{\left(\Omega \right)}^{d}}+\gamma \int_{\Omega }{\left|{\vec{v}}_{*}\right|}^{\beta -2}{\vec{v}}_{*}\cdot \vec{\eta }\;dx. \end{array}$$

Setting $\vec{\eta }={\vec{v}}_{*}$ in the last equality and applying integration by parts, we arrive at the energy equality (14).

To finish the proof of Theorem 1, it remains only to show that the statement (c) is true.

Clearly, we have

(49) $$\begin{array}{cc} \; & -\rho \sum_{i=1}^{d}\int_{\Omega }v_{\alpha_{n}i}{\vec{v}}_{\alpha_{n}}\cdot \frac{\partial \vec{\psi }}{\partial x_{i}}\;dx+\mu \int_{\Omega }D{\vec{v}}_{\alpha_{n}}:D\vec{\psi }\;dx-\alpha_{n}\sum_{i=1}^{d}\int_{\Omega }v_{\alpha_{n}i}D{\vec{v}}_{\alpha_{n}}:\frac{\partial D\vec{\psi }}{\partial x_{i}}\;dx\\ \; & =\rho {\langle \vec{F},\;\vec{\psi }\rangle }_{H^{-1}{\left(\Omega \right)}^{d}\times H_{0}^{1}{\left(\Omega \right)}^{d}}+\gamma \int_{\Omega }{\left|{\vec{v}}_{\alpha_{n}}\right|}^{\beta -2}{\vec{v}}_{\alpha_{n}}\cdot \vec{\psi }\;dx,\;\forall \;\vec{\psi }\in D_{\mathrm{sol}}{\left(\Omega \right)}^{d}, \end{array}$$

(50) $$\mu \int_{\Omega }|D{\vec{v}}_{\alpha_{n}}{|}^{2}\;dx\leq \rho {\langle \vec{F},\;{\vec{v}}_{\alpha_{n}}\rangle }_{H^{-1}{\left(\Omega \right)}^{d}\times H_{0}^{1}{\left(\Omega \right)}^{d}}+\gamma \int_{\Omega }{\left|{\vec{v}}_{\alpha_{n}}\right|}^{\beta }\;dx.$$

Arguing as in the proof of the statement (a), we can derive from (50) the estimate

$$∥{\vec{v}}_{\alpha_{n}}{∥}_{V^{1}\left(\Omega \right)}\leq C$$

with a constant *C* independent of $\alpha_{n}$. Moreover, without loss of generality it can be assumed that

(51) $$\begin{array}{cc} \; & {\vec{v}}_{\alpha_{n}}⇀{\vec{v}}_{0}\;\mathrm{weakly}\;\mathrm{in}\;V^{1}\left(\Omega \right)\;\mathrm{as}\;n\to \infty , \end{array}$$

(52) $$\begin{array}{cc} \; & {\vec{v}}_{\alpha_{n}}\to {\vec{v}}_{0}\;\mathrm{strongly}\;\mathrm{in}\;L^{s}{\left(\Omega \right)}^{d},\;s\in S\left(d\right),\;\mathrm{as}\;n\to \infty , \end{array}$$

(53) $$\begin{array}{cc} \; & |{\vec{v}}_{\alpha_{n}}{|}^{\beta -2}{\vec{v}}_{\alpha_{n}}\to {\left|{\vec{v}}_{0}\right|}^{\beta -2}{\vec{v}}_{0}\;\mathrm{strongly}\;\mathrm{in}\;L^{2}{\left(\Omega \right)}^{d}\;\mathrm{as}\;n\to \infty , \end{array}$$

for some vector function ${\vec{v}}_{0}\in V^{1}\left(\Omega \right)$.

Using (51)–(53) and the equality $\lim_{n\to \infty }\alpha_{n}=0$, we pass to the limit n→∞ in equality (49) and obtain:

$$\begin{array}{cc} \; & -\rho \sum_{i=1}^{d}\int_{\Omega }v_{0i}{\vec{v}}_{0}\cdot \frac{\partial \vec{\psi }}{\partial x_{i}}\;dx+\mu \int_{\Omega }D{\vec{v}}_{0}:D\vec{\psi }\;dx\\ \; & =\rho {\langle \vec{F},\;\vec{\psi }\rangle }_{H^{-1}{\left(\Omega \right)}^{d}\times H_{0}^{1}{\left(\Omega \right)}^{d}}+\gamma \int_{\Omega }{\left|{\vec{v}}_{0}\right|}^{\beta -2}{\vec{v}}_{0}\cdot \vec{\psi }\;dx,\;\forall \;\vec{\psi }\in D_{\mathrm{sol}}{\left(\Omega \right)}^{d}. \end{array}$$

This means that the vector function ${\vec{v}}_{0}$ is a weak solution of the steady Navier–Stokes equations with the damping term $\gamma |\vec{v}{|}^{\beta -2}\vec{v}$. Thus, Theorem 1 is completely proved.

## 6. Conclusions

In this paper, we studied the steady-state flow model for low-concentrated aqueous polymer solutions with a damping term in a bounded domain $\Omega \subset R^{d}$, d=2,3, subject to the no-slip condition on the boundary ∂Ω. Sufficient conditions for the existence of a full weak solution were established. Moreover, we derived the corresponding energy inequality and showed that solutions of the original problem converge to a solution of the steady-state damped Navier–Stokes system as the relaxation viscosity tends to zero. To obtain these results, we used the method of introduction of auxiliary viscosity, the acute angle theorem for ${\left(S\right)}_{+}$-operators, and the Krasnoselskii theorem on the continuity of the superposition operator in Lebesgue spaces. Note that all results were established for the essentially nonlinear system of partial differential equations without any simplifications of the flow model. The proposed approach is quite universal and provides ways for new investigations of such type models. The plan for future investigations includes the analysis of the well-posedness of nonlinear equations that describe time-dependent and/or temperature-dependent flows of aqueous polymer solutions with damping.

## Data Availability

Not applicable.
